# Supplementary material for: Therapeutic Landscapes and Psychiatric Care Facilities: A Qualitative Meta-Analysis
Source: Int J Environ Res Public Health. 2022 Jan 28;19(3):1490. doi: 10.3390/ijerph19031490 (PMC8835684; doi:10.3390/ijerph19031490)
Supplement: Supplementary file 1 [file ijerph-19-01490-s001.zip › ijerph-1540800-supplementary.pdf]

Table S1. Search strategies

Database	Number of results	Search string
MEDLINE (PubMed)	Initial search:1081<br>Alerts: 21	("Mental Health"[Title/Abstract] OR "Mental wellbeing"[Title/Abstract] OR "Mental Health Rehabilitation"[Title/Abstract] OR "Mental disorders"[Title/Abstract] OR "Stress"[Title/Abstract] OR "Mental health care"[Title/Abstract] OR "Neurological rehabilitation"[Title/Abstract] OR "Psychiatric Rehabilitation"[Title/Abstract] OR "Psychological distress"[Title/Abstract]) AND ("Health Facility Environment"[Title/Abstract] OR "Mental Health Service"[Title/Abstract] OR Hospital[Title/Abstract]) AND ("Therapeutic Landscapes"[Title/Abstract] OR "Therapeutic assemblage"[Title/Abstract] OR Gardening[Title/Abstract] OR "Therapeutic mobilities"[Title/Abstract] OR "Ecosystem services"[Title/Abstract] OR "Nature-based solutions"[Title/Abstract] OR "Healing Gardens"[Title/Abstract] OR "Green care"[Title/Abstract] OR Streetscape[Title/Abstract] OR "Green space"[Title/Abstract] OR "Blue Space"[Title/Abstract] OR Landscapes[Title/Abstract] OR Environment[Title/Abstract] OR "Virtual environment"[Title/Abstract] OR Horticulture[Title/Abstract] OR "Natural Resources"[Title/Abstract] OR "Neighborhood"[Title/Abstract] OR "Architecture"[Title/Abstract] OR "Healing Environment"[Title/Abstract] OR "Built environment"[Title/Abstract]) AND ((humans[Filter]) AND (english[Filter] OR german[Filter]) AND (2000:2021[pdat]))
PsycINFO	Initial search: 370<br>Alerts: 0	((((MA "mental health") OR ((TI "Mental wellbeing") OR (AB "Mental wellbeing")) OR ((TI "Mental Health Rehabilitation") OR (AB "Mental Health Rehabilitation")) OR (MA "stress") OR ((TI "mental health care") OR (AB "mental health care")) OR (MA "Mental Disorders") OR ((TI "Neurological rehabilitation") OR (AB "Neurological rehabilitation")) OR ((TI "Psychiatric Rehabilitation") OR (AB "Psychiatric Rehabilitation")) OR ((TI "Psychological distress") OR (AB "Psychological distress")))) AND ((("Health Facility Environment") OR (MA "Mental Health Services") OR (MA "Hospitals"))) AND ((("Therapeutic Landscapes") OR ("Therapeutic assemblage") OR ((TI "gardening") OR (AB "gardening")) OR ("Therapeutic mobilities") OR ((TI "Ecosystem services") OR (AB "Ecosystem services")) OR ((TI "Nature-based solutions") OR (AB "Nature-based solutions")) OR ((TI "Healing Gardens") OR (AB "Healing Gardens")) OR ("Green care") OR ((TI "Streetscape") OR (AB "Streetscape")) OR (TI "Green space" OR AB "Green space") OR ((TI "blue space") OR (AB "blue space")) OR ((TI "landscapes") OR (AB "landscapes")) OR (MA "Environment") OR ((TI "virtual environment") OR (AB "virtual environment")) OR ((TI "horticulture") OR (AB "horticulture")) OR "S30" OR (MA "Architecture") OR ((TI "Healing Environment") OR (AB "Healing Environment")) OR (MA "Built Environment") OR (MA "Natural Resources"))))<br>Limiters: Publication Year: 2000-2021, Population Group: Human
CINAHL	Initial search: 86<br>Alerts: 0	(((((MH "mental health")) OR ((TI "mental wellbeing") OR (AB "mental wellbeing")) OR ((TI "Mental Health Rehabilitation") OR (AB "Mental Health Rehabilitation")) OR ((MH "Stress")) OR (TI "mental health care" OR AB "mental health care") OR ((MH "Mental Disorders")) OR (TI "Neurological rehabilitation" OR AB "Neurological rehabilitation") OR (TI "Psychiatric Rehabilitation" OR AB "Psychiatric Rehabilitation") OR ((MH "Psychological Distress")))) AND ((((MH "Health Facility Environment")) OR ((MH "Mental Health Services")) OR ((MH "Hospitals")))) AND (((TI "Therapeutic Landscapes" OR AB "Therapeutic Landscapes") OR (TI "Therapeutic assemblage" OR AB "Therapeutic assemblage") OR (TI "Gardening" OR AB "Gardening") OR (TI "Therapeutic mobilities" OR AB "Therapeutic mobilities") OR (TI "Ecosystem services" OR AB "Ecosystem services") OR (TI "Nature-based solutions" OR AB "Nature-based solutions") OR (TI "Healing Gardens" OR AB "Healing Gardens") OR (TI "Green care" OR AB "Green care") OR (TI "Streetscape" OR AB "Streetscape") OR (TI "Green space" OR AB "Green space") OR (TI "Blue Space" OR AB "Blue Space") OR (TI "Landscapes" OR AB "Landscapes") OR ((MH "Environment")) OR (TI "Virtual environment" OR AB "Virtual environment") OR ((MH "Horticulture")) OR (TI "neighborhood" OR AB "neighborhood") OR ((MH "Architecture")) OR (TI "Healing Environment" OR AB "Healing Environment") OR ((MH "Built Environment")) OR (TI "Natural Resources" OR AB "Natural Resources"))))<br>Limiters: Published Date: 20000101-20211231

(table continues)

Table S1. *(continued)*

Web of Sciences (Expanded)	Initial search: 1263<br>Alerts: 31	(TS= ("Mental Health" OR "Mental wellbeing" OR "Mental Health Rehabilitation" OR "Mental disorders" OR Stress OR "Mental health care" OR "Neurological rehabilitation" OR "Psychiatric Rehabilitation" OR "Psychological distress") AND TS=("Health Facility Environment" OR "Mental health service" OR "Hospitals") AND TS= ("Therapeutic Landscapes" OR "Therapeutic assemblage" OR Gardening OR "Therapeutic mobilities" OR "Ecosystem services" OR "Nature-based solutions" OR "Healing Gardens" OR "Green care" OR Streetscape OR "Green space" OR "Blue Space" OR Landscapes OR Environment OR "Virtual environment" OR Horticulture OR Neighborhood OR Architecture OR "Healing Environment" OR "Built environment" OR "Natural Resources")) *AND* LANGUAGE: (English OR German) *AND* DOCUMENT TYPES: (Article)<br>Timespan: 2000-2021. Indexes: SCI-EXPANDED, SSCI, A&HCI, CPCI-S, CPCI-SSH, ESCI, CCR-EXPANDED, IC

Table S2. Overview of the key themes, definition of themes, and supporting quotes

Key theme	Definition	Subtheme	Supporting quotes from the original articles
Physical dimension			
Design features	Description of regular design (elements) of a facility, such as furniture or spatial distribution	Amenity	“A room of your own or at least your own space with a bed, a bedside table and a wardrobe. (Interviewee 1)” (Schröder and Ahlström 2004 p. 8)
		Space	“I think this overall objective they had for nurses and clinicians to be more interactive with the patients rather than staying in the nursing station all day is kind of unrealistic, not saying that nurses should be encouraged to stay in the nursing station all day, but it’s just this daily reality that patients don’t want nurses around them every day. (FG4)” (Novotná et al. 2011 p. 1533)
		Economic benefits	Quantitative study data, no supportive quote available (see Nanda et al. 2011 pp. 391–392)
Perception (of the physical environment)	Perception of specific design elements and their attributed effects	Comfort	"I felt quite low about myself and the surroundings at ****'s are very low and so I felt that I fitted in at first." (Gilburt et al. 2008 results section, “Environment” paragraph, second quote)
		Staffs view on patient outcome	“[Outdoor spaces] needs to be very usable. I believe those gardens should have places for sports and recreation and vegetable gardens. They should attract birds and butterflies. Because those are the things that make the place joyful and those are the kinds of features that I believe make people better.” (Shepley et al. 2016 p. 17)
Social dimension			
Features of the social dimension	Impact of the environment on human-environment interaction and interaction/relationship between different stakeholder groups	Confusing space	“She added, ‘to me it seems like a confused space, and people don’t know what to do in that space outside of meal times, other than just sit there’.” (Donald et al. 2015 p. 65)
		Safe space	“A man was in my room, sitting on my bed touching everything. I told him to get out and he refused. I was scared and mad. They [staff] couldn’t get him out. They had to call security. I was so upset. I feel my privacy has been violated. They gave me pills to calm down but I couldn’t sleep all night.” (Hung et al. 2014 p. 5)
		Encouraging/ discouraging space	“Some reasons (for absconding) are probably the feeling of being powerless. To feel that somebody has total control over what you do, when you shower, when you go to bed, when you’re allowed out for a smoke has a massive effect on me, massive effect. (Consumer 7.)” (Muir-Cochrane et al. 2013 p. 309)

		Social connection	"The outside garden area was good...you could go for a cigarette and sit outside and talk to the other patients. We all ended up as friends, most of the patients, but yeah it was really nice, you felt secure as well as safe [Discharged patient (acute) 1]." (Wood et al. 2013a p. 110)<br><br>"One member of staff suggested that when patients and staff could smoke together '*the patient would actually sit and talk to you, they would see you on the same level then'*; in the same discussion, we were also told that '*in the past you always got a lot more information out of patients in the smoke room, having a cigarette, when you could sit with them*' [Forensic ward Staff 2]." (Wood et al. 2013a p. 110)

(table continues)

Table S2. *(continued)*

Symbolic environment			
(Therapeutic) Value	The emerging therapeutic value through aspects of TL in a facility	Facilitators	''I also explored my artistic side here, and I realized that I liked it a lot, and this was very important for me.'' (Agrest et al. 2018 p. 3)
		Barriers	"Susan said she would be 'glad to see the place [Old Hospital] knocked down' because it 'reinforces the whole stigma attached to mental health'." (Wood et al. 2013b p. 125)
